# Effect of Different Exercise Intensities on Hepatocyte Apoptosis in HFD-Induced NAFLD in Rats: The Possible Role of Endoplasmic Reticulum Stress through the Regulation of the IRE1/JNK and eIF2*α*/CHOP Signal Pathways

**DOI:** 10.1155/2021/6378568

**Published:** 2021-03-15

**Authors:** Ling Ruan, Fanghui Li, Shoubang Li, Mingjun Zhang, Feng Wang, Xianli Lv, Qin Liu

**Affiliations:** ^1^Department of Physical Education, Xi'an Shiyou University, Xi'an, Shaanxi, China; ^2^School of Sports Sciences, Nanjing Normal University, Nanjing, Jiangsu, China; ^3^School of Sports and Health Sciences, Xi'an Physical Education University, Xi'an, Shaanxi, China; ^4^Ankang Traditional Chinese Medicine, Ankang, Shaanxi, China; ^5^School of Physical Education, Ankang University, Ankang, Shaanxi, China

## Abstract

**Objective:**

To investigate the impact of different-intensity exercise on lipid metabolism, oxidative stress, hepatocyte injury, and apoptosis and the related protein expression of endoplasmic reticulum stress on nonalcoholic fatty liver disease rats.

**Method:**

50 male Sprague–Dawley rats, 2 months old, were randomly divided into the normal control (CON) group, high-fat diet (HFD) group, low-intensity exercise (LIE) group, moderate-intensity exercise (MIE) group, and incremental-intensity exercise (IIE) group. Blood lipids were tested by the automatic biochemical analyzer. The changes in liver tissues were observed by hematoxylin-eosin staining (HE). The protein expression of Bax and Bcl-2 was detected by the immunohistochemical method. The apoptosis of hepatocytes was detected by the TUNEL method. The protein expression of GRP78, Caspase-3, IRE1, p-IRE1, JNK1, CHOP, PERK, eIF2*α*, and ATF4 was detected by Western blotting.

**Results:**

Our study showed that compared with the HFD group, TG, TC, FFA, and LDL-c were reduced in all exercise groups. The different exercise intensities could reduce the protein expression of ATF4, Bax, and hepatocyte apoptosis. Meanwhile, the antioxidant function and Bcl-2 were increased. However, the moderate-intensity exercise demonstrated more effect on improving the antioxidant capacity and inhibiting hepatocyte apoptosis. Compared with the HFD group, Caspase-3 and JNK were significantly decreased in all exercise groups (*P* < 0.01) and CHOP was decreased in the LIE and MIE groups (*P* < 0.05). IRE1, eIF2*α*, the ratio of p-IRE1/IRE1 (*P* < 0.01), and ATF4 were decreased (*P* < 0.05) in the MIE group. Compared with the IIE group, p-IRE1 was decreased (*P* < 0.05) in the MIE group. GRP78 had no significant difference among the exercise groups.

**Conclusion:**

Exercise at different intensities improved blood lipid and hepatic injury in NAFLD rats. However, the body weight of the rats in each exercise group was not significantly different. Moderate-intensity exercise demonstrated more effect on improving the antioxidant ability and inhibiting hepatocyte apoptosis. The possible mechanism depends on the regulation of endoplasmic reticulum stress signaling pathways IRE1/JNK and eIF2*α*/CHOP.

## 1. Introduction

Nonalcoholic fatty liver disease (NAFLD) is the hepatic manifestation of metabolic syndrome, and it is the most prevalent liver disease worldwide [1]. The prevalence of NAFLD is approximately 30% in the United States and Europe, with a similar prevalence documented in Asian countries [2]. It encompasses a spectrum ranging from simple steatosis to fatty liver with hepatocellular injury, termed nonalcoholic steatohepatitis (NASH), fibrosis, and cirrhosis [3].The pathological mechanisms of NAFLD development are primarily ascribed to lipid metabolism disorders, oxidative stress, and hepatocyte apoptosis, as interpreted by the “two-hit hypothesis” [4, 5]. Emerging evidence suggests that hepatocyte apoptosis may be a key component of the “second hit” involved in the progression of NAFLD [6], especially that, during the “second hit”, hepatocytes suffer from oxidative stress and lipid peroxidation, resulting in hepatocyte apoptosis [7, 8]. Meanwhile, reducing the number of hepatocyte apoptosis may be a novel therapeutic target for regulating NAFLD development [9].

Several pharmacological and nonpharmacological strategies have been proposed to relieve NAFLD-associated deleterious alterations [10]. Among nonpharmacological approaches, physical exercise-mediated multisystemic adaptations and promoting a crosstalk between organs and orchestrating the prometabolic effects are known to mitigate metabolism-related disorders such as NAFLD [11]. Keating et al. [12] examined the efficacy of the commonly prescribed exercise dose and intensity for reducing liver fat and visceral adipose tissue in an experimental animal model of NAFLD, but no significant differences were found between the dose or intensity of the exercise regimen and reductions in liver fat or visceral adipose tissue. Paradoxically, it has been shown that vigorous and moderate exercises were equally effective in reducing the intrahepatic triglyceride content [13, 14] but body weight, body fat, waist circumference, and blood pressure in the vigorous-moderate exercise group were lower compared with those in the moderate exercise group. Similarly, Tsunoda et al. [14] have reported that vigorous-intensity exercise was more effective than moderate low-intensity exercise and the moderate high-intensity protocol in preventing nonalcoholic simple fatty liver from progressing to NASH. Two systematic reviews of published studies of NAFLD patients participating in aerobic exercise programs showed that liver fat was significantly reduced but the optimal exercise intensity is undetermined [15, 16], although a growing body of prospective data shows the effects of different types of exercise on NAFLD [17]. Collectively, those previous ones suggest that the intensity of exercise, rather than its volume or duration, may play a critical role in magnifying the protective effect of exercise against NAFLD [18].

The biological mechanisms underlying the effect of exercise on NAFLD remain poorly understood. A previous study demonstrated that endurance exercise reduces a circulating marker of hepatocyte apoptosis in previously sedentary obese individuals with NAFLD and reductions in apoptosis may be related to the fat oxidation capacity [6]. Moreover, Goncalves et al. [10] showed that endurance exercise prevented the release of cytochrome c from mitochondria, attenuated potential membrane dissipation, and therefore mitochondrially drove apoptotic signaling in hepatocyte from the HFD-induced NAFLD rat model. However, the role and underlying mechanism of exercise training in hepatocyte apoptosis remain poorly understood.

One potential mechanism relates to obesity-induced stress on the endoplasmic reticulum (ER), which is an essential intracellular organelle responsible for folding and modifying. Disruption of either protein folding or changing within the ER activates the unfolded protein response (UPR), which conversely promotes cell death by activating downstream apoptosis molecules if ER stress is prolonged and severe. Recent studies have shown that hepatic ER stress seems to play a central role in the mechanism that relates to NAFLD-induced hepatocyte apoptosis [19]. Furthermore, proapoptotic molecules such as eukaryotic translation initiation factor 2*α* (eIF2*α*), glucose-regulated protein 78 (GRP78), inositol-requiring enzyme 1*α* (IRE1*α*), ER-resident PKR-like eIF2*α* kinase (PERK), C/EBP homologous protein (CHOP), cleaved Caspase-3, and JNK were detected and JNK activation and CHOP expression via ER stress-mediated proapoptotic cascades occurred prior to Caspase-3 activation in high-fat diet-fed-induced NAFLD mice compared with rats that were fed a standard chow diet [20, 21].

In this study, we hypothesize that oxidative stress and ER stress-mediated proapoptotic cascades in hepatocyte may be involved in the protective effect of exercise intensity dependence against NAFLD. We tested this hypothesis by determining metabolic as well as hepatic oxidative stress and hepatocyte apoptosis and markers of ER stress protein expression in HFD-induced NAFLD rats to low-intensity endurance exercise (LIE) and middle-intensity endurance exercise (MIE) and incremental-intensity exercise (IIE).

## 2. Materials and Methods

### 2.1. Animals

Male 8-week-old Sprague–Dawley rats ranging from 180 to 220 g were obtained from the Guangdong Medical Laboratory Animal Center (GDMLAC) (Guangzhou, China), according to the principles of the Declaration of Helsinki, and the experiments were approved by the Animal Experiment Ethical Inspection Form of Huanan Normal University (IACUU-2008-0020).

The animals were housed in their respective groups in a collective cage and received water and standard laboratory chow (56.8% carbohydrate, 22.5% protein, 3.5% lipids, and 17.2% other nutrients) ad libitum. The high-fat diet (HFD) (GDMLAC) contained several compounds that provide energy (consisting of 18% lard, 5% sucrose,15% egg yolk powder, 0.5% sodium cholate, 1% cholesterol, and 60.5% basic forage). Fifty experimental rats were randomly divided into five groups: (1) the normal control (CON) (*n* = 12) group, (2) HF diet (HFD) (*n* = 10) group, (3) HFD & low-intensity exercise (LIE) group, (4) HFD & moderate-intensity exercise (MIE) group, and (5) HFD & incremental-intensity exercise (IIE) group. Rats were maintained on a 12 h light/dark cycle and fed ad libitum with HFD or standard chow diet for 16 weeks starting at 8 weeks of age. After the 16th week of HFD, rats were either exercise trained or remained sedentary for the final 7 weeks (including being familiarized with treadmill running for one week). At the end of the experiment, blood and liver samples were harvested for analysis. All the procedures were performed in compliance with the institute's guidelines and with the Guide for the Care and Use of Laboratory Animals. The study was approved by the institutional animal care committee of GDMLAC.

### 2.2. Experimental Protocol

All animals were familiarized with treadmill running (DSPT202, Qian Jiang Technology Company, Hangzhou, China) at 0–15 m/min, 10–20 min per day for five consecutive days. An electrified grid (0.6 mA intensity) was placed behind the belt of the treadmill to induce running. The rats that failed to run regularly were excluded from the training protocol. The exercise program involved 5 sessions per week for 6 weeks. Every training session began with a10-min warm-up (10 m/min), and the speed was then progressively increased for 60 min to the appropriate speed for each training group. The LIE group consisted of running at a speed of 15 m/min for 6 weeks at a 0° inclination (about 30% maximal oxygen consumption). The first week of the MIE group training program was 60-min running at the speed of 20 m/min, then running 60 minutes at the speed of 25 m/min in the following 5 weeks.. The training program for the IIE group consisted of running for 40 minutes at 20 m/min and 20 minutes at 22 m/min in the first week, inclined at 10°; the second week consisted of running for 40 minutes at 25 m/min and 20 minutes at 27 m/min. During the third week, running training intensity was progressively increased to 30 min at 25 m/min and 30 min 28 m/min, inclined at 10°. Finally, for the remaining 3 weeks, exercise was performed for 30 min at 25 m/min and 30 min at 30 m/min (about 75% maximal oxygen consumption). Six animals were excluded from the study; 4 rats (2 MIE, 1 IIE, and 1 HFD) died (exercise-unrelated causes) before the end of the study, 1 rat from the LIE group was excluded for not finishing the training plan, and 1 rat from the MIE group was excluded due to severe eye infections (two rats fighting and one of them got injured).

### 2.3. Sample Preparation

The rats underwent fasting for 12 hours and were drinking freely after the last training. Anesthetized with 3% pentobarbital on the next day, blood samples were obtained from the abdominal cavity, the liver was quickly dissected from the largest lobe, and 2 pieces of liver tissue (2 cm × 1 cm × 0.3 cm) were cut out and fixed with 4% paraformaldehyde. The remaining liver tissue is frozen in liquid nitrogen and stored in a refrigerator at −80°C.

### 2.4. Serum Analysis

Blood samples (5 ml) were obtained from the abdominal cavity and centrifuged at 3000 rpm for 15 min, and then, serum was collected. The serum triglyceride (TG) levels (mmol/l), total cholesterol (TC) levels (mmol/l), low-density lipoprotein cholesterol (LDL-c) levels (mmol/l), high-density lipoprotein cholesterol (HDL-c) levels (mmol/l), blood glucose levels (mg/dl), alanine aminotransferase (ALT) levels (U/l), and aspartate transaminase (AST) levels (U/l) were measured according to the manufacturer's protocols (Nanjing Jian Cheng Corp., Nanjing, China).

### 2.5. Hepatic Oxidative Stress

Liver tissue (200 mg) taken from the same part of the liver were tested by hepatic oxidative stress. Hepatic malondialdehyde (MDA) levels were quantified using a lipid peroxidation MDA assay kit (Beyotime Institute of Biotechnology, Jiangsu, China) according to the manufacturer's protocol. Hepatic superoxide dismutase (SOD) activity (Superoxide Dismutase Assay Kit (WST-1 method)), catalase (CAT) activity, (Catalase (CAT) Assay Kit (visible light method)), glutathione s-transferase (GST) levels, and total antioxidant capacity (T-AOC) (Total Antioxidant Capacity Assay Kit (ABTS method)) were determined using a reagent kit (Nanjing Jian Cheng Corp.). The results were corrected for protein content.

### 2.6. Hematoxylin-Eosin (HE) Staining

Liver tissue (2 cm × 1 cm × 0.3 cm) was fixed with 10% formalin and cut into 10 *μ*m sections with a cryostat for hematoxylin-eosin staining, and then, at least three randomly selected liver sections from each group were examined and photographed with a Nikon Eclipse Ci light microscope (Nikon Corp., Tokyo, Japan).

### 2.7. TUNEL Assay and Apoptotic Markers in the Liver Tissue

Liver tissue samples were fixed in neutral-buffered formalin (10%), embedded in paraffin, and sectioned into 4 *μ*m sections. Terminal deoxynucleotidyl transferase-mediated dUTP nick end labeling (TUNEL) assay was performed with an In Situ Cell Death Detection Kit (TMR Red; Roche, NJ, USA) according to the manufacturer's instructions. Then, we identified positive staining in the cell nucleus with DNA breakage under the light microscope (Nikon Corp., Tokyo, Japan).

Bcl-2, Bax, and ATF4 protein expression was determined using immunohistochemistry reactions, and they were performed using the peroxidase/anti-peroxidase (PAP) method. Nonspecific peroxidase reactions were blocked with methanol containing 0.1% hydrogen peroxide. The sections were incubated with normal goat serum to avoid nonspecific reactions once the samples were incubated with specific antibodies against Bcl-2, Bax, and ATF4 (dilution 1 : 2000, Santa Cruz CA, USA). Tissue sections were then washed with phosphate buffer and incubated with secondary antibodies (1 : 2000; Sigma, USA), before being rewashed in phosphate buffer and incubated with the PAP complex (dilution 1 : 200). The peroxidase reaction was carried out using a solution of 3,3′-diaminobenzidine tetrahydrochloride containing 0.01% hydrogen peroxide in Tris-HCl buffer (0.05 M, pH 7.6). After immunostaining, the liver sections were lightly counterstained with hematoxylin and observed under a light microscope [22].

### 2.8. Western Blot Analysis

1 ml of RIPA, 10 *μ*l 100 mM of PMSF, and 10 *μ*l of phosphatase inhibitors were added into every 100 mg of liver tissue. After homogenizing, the sample was centrifuged at high speed; the supernatant was carefully transferred into a clean sterile centrifuge tube and stored in a refrigerator at −20°C. The liver tissue lysate was taken and the sample buffer was added to electrophoresis (5% concentrated gel 90 V, 12% separated gel 110 V, and unsteady current) for about 1.5 h. The separation gel was transferred to membrane (90 V, 90 min), and then, the electric rotation instrument was turned on. The nitrocellulose membrane (NC) was taken out and washed with deionized water. After the washing, it was sealed with 5% skimmed milk powder (TBS) at indoor temperature for 30 min and then was stored at 4°C overnight. Added the diluted mouse and rabbit anti (GRP78, Caspase-3, p-IRE1, JNK1, CHOP, PERK, eIF2*α*) (Santa Cruz,USA), overnight at 4°C , the membrane was washed with PBS, and then added 5ml 1:5000 dilution (the dilution contains 2% skimmed milk powder) goat anti rabbit second antibody(Bejing Boason), reaction time was 1h at 37°C. Sucked up the PBS of the NC, then added ECL chemical luminescent reagent, after completely soaking, put it on the film, cover the fresh-keeping film, put it into the X-ray cassette, use the Quantity-one gel electrophoresis image software system to analyze.

Nuclear and cytosolic proteins were extracted from liver tissues with a protein extraction kit (KeyGen Biotech, Nanjing, China), and cells were lysed with RIPA buffer. Equal amounts of protein from each sample were separated by 10–15% SDS-PAGE (Bio-Rad, Hercules, USA). Blots were incubated overnight at 4°C with the following primary antibodies: GRP78, Caspase-3, CHOP, IRE1, p-IRE1, PERK, eIF2*α*, JNK1, and *β*-actin (Santa Cruz Biotechnology, Santa Cruz, CA, USA). The blots were then immune stained with secondary antibodies at 37°C. The membranes were exposed to enhanced Chemiluminescence Reagents Plus (Beyotime Institute of Biotechnology). The images were captured using a BioSpectrum 410 Multispectral Imaging system and analyzed with a Gel-Pro Analyzer (version 5.0; Media Cybernetics, Rockville, MD, USA).

### 2.9. Characterization of Nonalcoholic Fatty Liver Disease

To characterize NAFLD, paraffin-embedded liver tissue samples were cut into 5 *μ*m thick sections for hematoxylin and eosin (HE) staining and the sections were then examined by light microscopy. Rat livers were fixed with 10% formalin, cut into 10 *μ*m sections with hematoxylin and eosin, and then, at least three randomly selected liver section images which were digitally captured (400x magnification), from each group, were examined and photographed with a Nikon Eclipse Ci light microscope (Nikon Corp., Tokyo, Japan). The pathological section of liver tissue was sent to two professional pathologists for double-blind diagnosis. Histopathology scoring was evaluated in terms of the degree of steatosis (score 3), lobular inflammation (score 3), ballooning (score 2), and liver fibrosis (score 0), according to the system proposed by Kleiner [23].

### 2.10. Statistical Analysis

Data are presented as mean ± SD. GraphPad Prism (version 5.0; GraphPad Prism Software, La Jolla, CA, USA), one-way ANOVA, and Tukey's honestly significant difference post hoc analysis were conducted to establish significant differences. Statistical analyses were performed using differences which were considered as statistically significant at *P* < 0.05. Pearson's correlation coefficient was employed to analyze the correlations between parametrical data.

## 3. Results

### 3.1. Effect of Different Exercise Training Intensities on Food Intake

**Figure 1:**
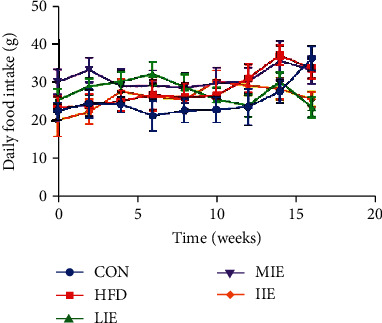
Effects of exercise training on food intake. Change of food intake in rats fed a standard chow diet or HFD and treated with different-intensity exercise.

As shown in Figure 1, there was no significant difference in food intake of rats fed a standard chow diet or HFD groups during the experiment procedure.

### 3.2. Effect of Different Exercise Training Intensities on Body Weight and Liver Weight

**Figure 2:**
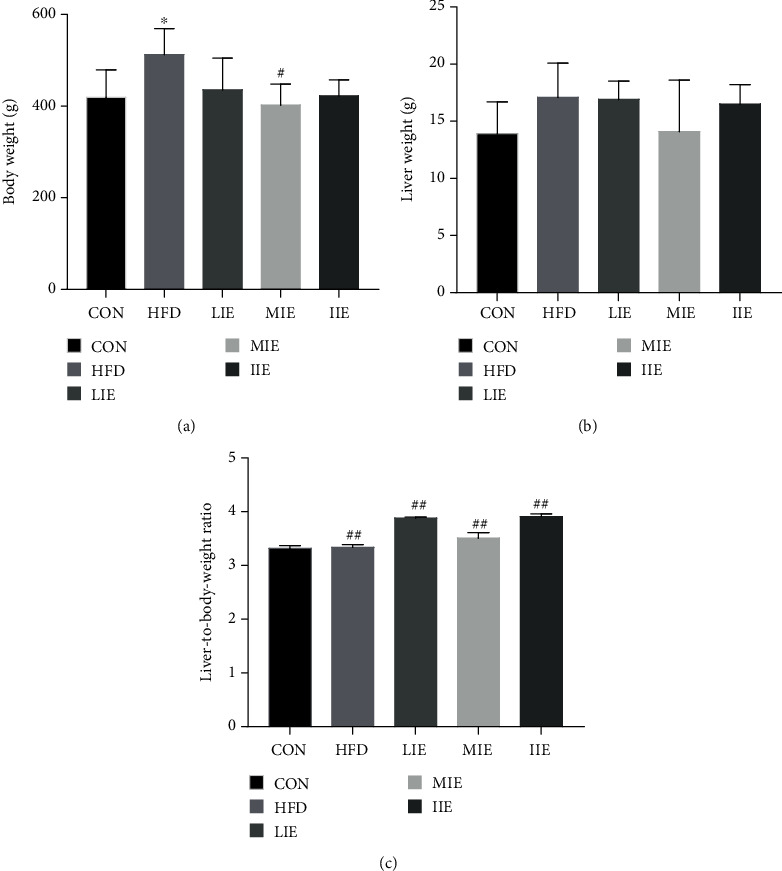
Effects of exercise training on body weight, liver weight, and the liver-to-body-weight ratio. Changes of (a) body weight, (b) liver weight, and (c) liver-to-body-weight ratio of rats fed a standard chow diet or HFD and treated with different-intensity exercise. ^∗^*P* < 0.05 vs. CON; ^#^*P* < 0.05 and ^##^*P* < 0.01 vs. HFD. All data are expressed as mean ± SD; 6–8 animals per group were used.

Changes observed in body weight, liver weight, and the liver-to-body-weight ratio are presented in Figure 2. At the end of this experiment, the body weight (*P* < 0.05) of the HFD group had increased compared with that of the CON group but no significant decreases in the body weight and liver weight were identified in all the exercise intervention groups compared with the HFD group (*P* > 0.05).

### 3.3. Effect of Different Exercise Training Intensities on Lipid Metabolism Disorders and Liver Injury

**Figure 3:**
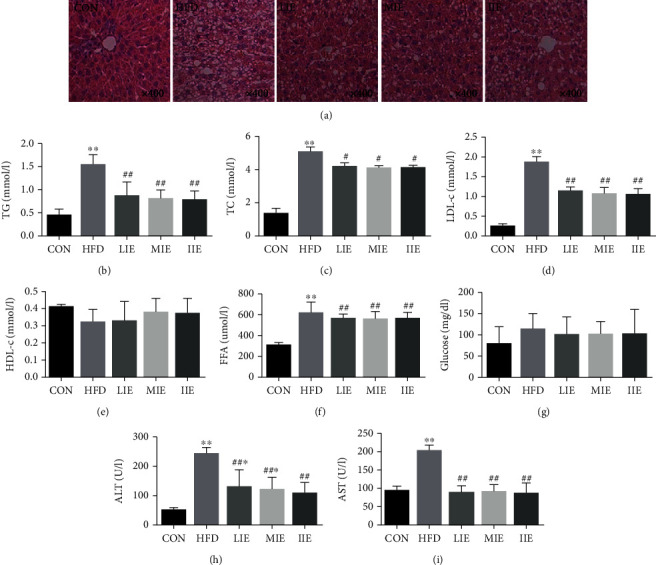
Effect of different exercise training intensities on lipid metabolism disorders and liver injury. Representative photomicrographs of hematoxylin and eosin- (HE) stained liver sections of CON, HFD, LIE, MIE, and IIE (magnification ×400) (a). The change of serum TG (c), TC (d), LDL-c (e), HDL-c (f), FFA (g), ALT (h), and AST (i) in each group; ^∗^*P* < 0.05 and ^∗∗^*P* < 0.01 vs. CON; ^#^*P* < 0.05 and ^##^*P* < 0.01 vs. HFD. All data are expressed as mean ± SD; 6–8 animals per group were used.

HFD was fed to rats to establish a NAFLD model, and the phenotypes were observed following treatment with different exercise intensities (Figure 3). Liver histology was evaluated by hematoxylin-eosin (HE) staining. The CON group exhibited well-arranged hepatic cords, cells with round and central nuclei, a lobular structure, and an array of wheel-shaped cells along the centrilobular vein. However, in the HFD group, lipid droplets were observed in the liver sections (Figure 3(a)). Lipid droplet volumes and quantities were reduced by treatment with different exercise intensities. These findings demonstrated that feeding HFD to rats was able to successfully establish a model of disordered lipid metabolism. Furthermore, different exercise training intensities improved this lipid metabolism disturbance.

Selected lipid metabolism parameters were detected in the four experimental groups (Figures 3(b)–3(f)). At the end of the experiment, a significant increase in serum TC, TG, FFA, and LDL-c levels (*P* < 0.01) was identified in the HFD group compared with the CON group, whereas HDL-c levels were unaltered (*P* > 0.05). Significant decreases in serum TC (*P* < 0.05), TG (*P* < 0.01), FFA (*P* < 0.01), and LDL-c levels (*P* < 0.01) were identified in the LIE, MIE, and IIE intervention groups compared with the HFD group. There was no significant difference in serum HDL-c following these three different-intensity exercise treatments (*P* > 0.05). Notably, the majority of lipid metabolism parameters exhibited being equally effective between these three exercise training intensity groups.

Compared with the CON group, the HFD-fed rats were characterized by significantly higher serum ALT and AST activities (*P* < 0.01; Figures 3(h) and 3(i)). However, following three different exercise training intensities, ALT and AST activities significantly reduced (*P* < 0.01). These findings also suggested that lipid metabolism disorders and hepatocellular damage were present in the rat model and that exercise training was able to correct them.

### 3.4. Effect of Different Exercise Training Intensities on HFD-Induced Liver Oxidative Stress

**Figure 4:**
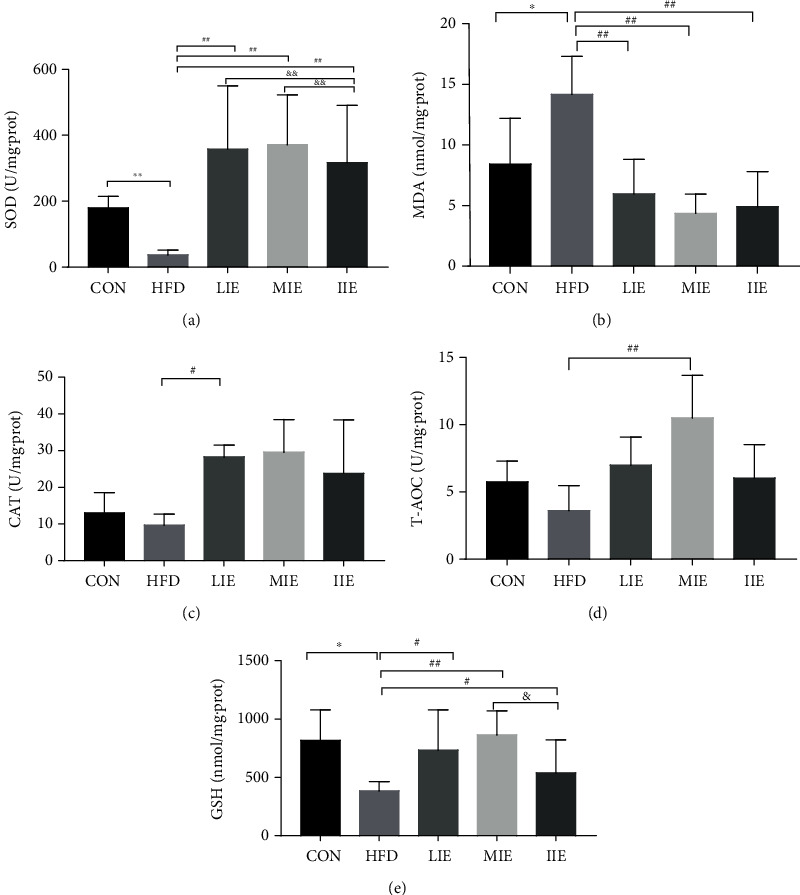
Effect of different exercise training intensities on markers of oxidative status in the liver tissue. MDA (a), CAT (b), GST (c), SOD (d), and T-AOC (e) of rats fed a standard chow diet or HFD and treated with different exercise intensities. MDA: malondialdehyde; CAT: catalase; GST: glutathione s-transferase; SOD: superoxide dismutase; T-AOC: total antioxidant capacity; ^∗^*P* < 0.05 and ^∗∗^*P* < 0.01 vs. CON; ^#^*P* < 0.05 and ^##^*P* < 0.01 vs. HFD; ^&^*P* < 0.05 and ^&&^*P* < 0.01 vs. IIE. All data are expressed as mean ± SD; 6–8 animals per group were used.

The effects of 6 weeks of exercise intervention on oxidative stress parameters are presented in Figure 4. The present study shows that HFD-induced oxidative stress in the liver as indicated by a significant (*P* < 0.01) decline in enzymatic SOD (179.4 ± 10.1 at CON vs. 37.3 ± 3.3 U/mg·prot at HFD, Figure 4(a)), no significant decline in CAT (13.05 ± 5.52 at CON vs. 9.72 ± 3.0 U/mg·prot at HFD, Figure 4(c)), T-AOC activity (8.75 ± 1.55 at CON vs. 3.62 ± 1.85 U/mg·prot at HFD, Figure 4(d)), MDA concentration (8.43 ± 5.2 at CON vs. 14.2 ± 6.0 nmol/mg·prot at HFD, Figure 4(b)), and nonenzymatic antioxidant molecule GSH activity (818.35 ± 261.62 at CON vs. 384.32 ± 80.45 U/mg·prot at HFD, Figure 4(e)) was found when compared with that in the CON group.

All of the exercise groups had significantly higher SOD activity (LIE vs. HFD, *P* < 0.01; MIE vs. HFD, *P* < 0.01; and IIE vs. HFD, *P* < 0.01) and lower MDA concentration (LIE vs. HFD, *P* < 0.05; MIE vs. HFD, *P* < 0.01; and IIE vs. HFD, *P* < 0.01) in liver tissue compared with the HFD group (*P* < 0.01), LIE group, and MIE group which had significantly higher SOD activity compared with the IIE (LIE vs. IIE, *P* < 0.01 and MIE vs. IIE, *P* < 0.01) groups. Moreover, a significant increase in liver CAT (*P* < 0.05) and T-AOC activities (*P* < 0.01) and GSH activity (*P* < 0.05) was only identified in the MIE group compared with the HFD group.

### 3.5. Effect of Different Exercise Training Intensities on HFD-Induced Hepatocyte Apoptosis

**Figure 5:**
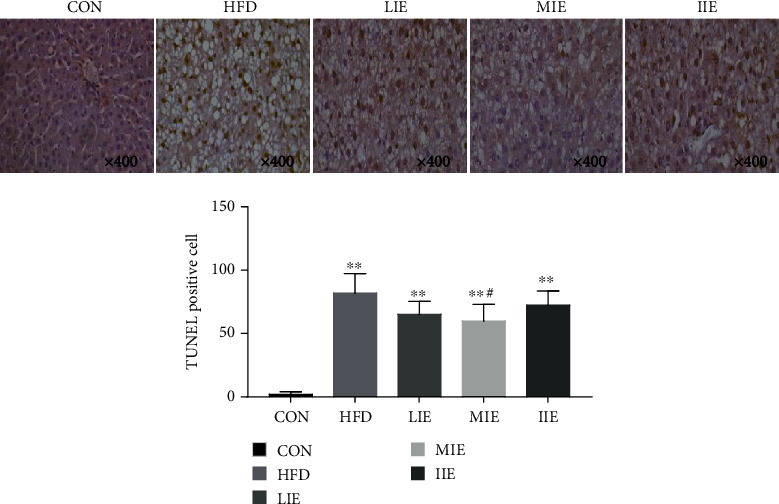
The positive expression of apoptosis cells in liver tissue by TUNEL assay. Terminal deoxynucleotidyl transferase-mediated UTP nick end labeling (TUNEL). ^∗∗^*P* < 0.01 vs. CON; ^#^*P* < 0.05 vs. HFD. All data are expressed as mean ± SD; 6–8 animals per group were used.

The effects of HFD and 6 weeks of different exercise training intensities on hepatocytes apoptosis in different groups were summarized in Figure 5. The present study shows that HFD-induced hepatocyte apoptosis in the liver as indicated by a significant (*P* < 0.01) increase in TUNEL positive staining in cell nucleus was found when compared with the CON group but the hepatocyte apoptosis was decreased by 21.5% (*P* > 0.05), 25.5% (*P* < 0.05), and 12.3% (*P* > 0.05)in the LIE, MIE, and IIE groups, respectively, as compared with the HFD group.

### 3.6. Effect of Different Exercise Training Intensities on Bax and Bcl-2 Protein Expression

**Figure 6:**
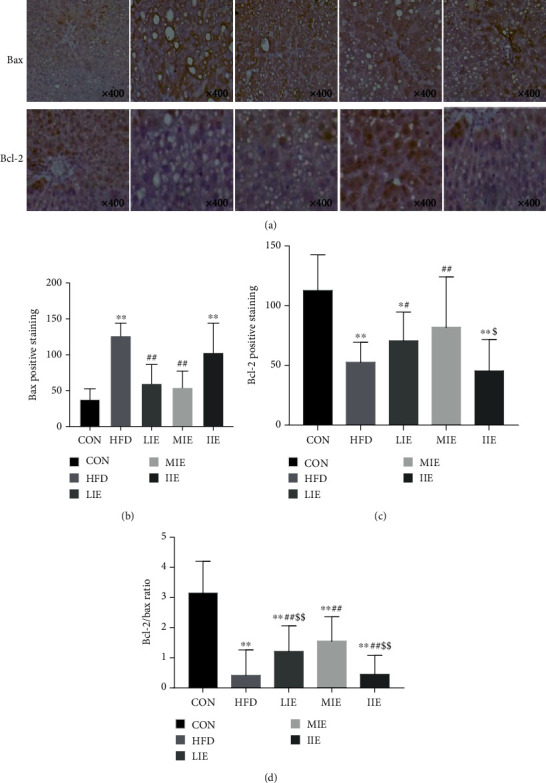
Immunohistochemical staining analyses of Bax, Bcl-2, and the Bcl-2/Bax ratio. Representative photomicrographs of immunohistochemical staining for Bax and Bcl-2 (a) protein expression (magnification ×400). Changes in Bax (b) and Bcl-2 (c) protein expression and the Bcl-2/Bax ratio (d) in each group; ^∗^*P* < 0.05 and ^∗∗^*P* < 0.01 vs. CON; ^#^*P* < 0.05 and ^##^*P* < 0.01 vs. HFD; ^$^*P* < 0.05 and ^$$^*P* < 0.01 vs. MIE. All data are expressed as mean ± SD; 6–8 animals per group were used.

Figure 6 shows the alteration of Bax and Bcl-2 protein expression and the Bcl-2/Bax ratio in the experimental groups. Bcl-2 protein expression (*P* < 0.01) and the Bcl-2/Bax ratio (*P* < 0.01) in the liver were significantly decreased by 53.4% and 638.7%, respectively, in the HFD group after 8 weeks while Bax (*P* < 0.01) protein expression was significantly increased by 250% in the HFD group, as compared with the CON group.

Bax protein expression was increased by 53.6% (*P* < 0.01), 57.8% (*P* < 0.01), and 18.8% (*P* > 0.05) in the LIE, MIE, and IIE groups, respectively, in comparison with the HFD group. Bcl-2 protein expression was increased by 34.3% (*P* < 0.05) and 56.1% (*P* < 0.01) in the LIE and MIE groups, respectively, in comparison with the HFD group. The Bcl-2/Bax ratio was significantly increased by 188% (*P* < 0.01) and 269% (*P* < 0.01) in the LIE and MIE groups, respectively, in comparison with the HFD group. Moreover, Bcl-2 protein expression (*P* < 0.05) in the MIE group significantly increased, as compared with the IIE group (*P* < 0.05), while the Bcl-2/Bax ratio in the MIE group was considerably higher than those in the LIE and IIE groups (*P* < 0.01).

### 3.7. Exercise Training Decreased the HFD-Induced Hepatic GRP78, Caspase-3, and PERK-eIF2*α*-CHOP Pathways in Rats

**Figure 7:**
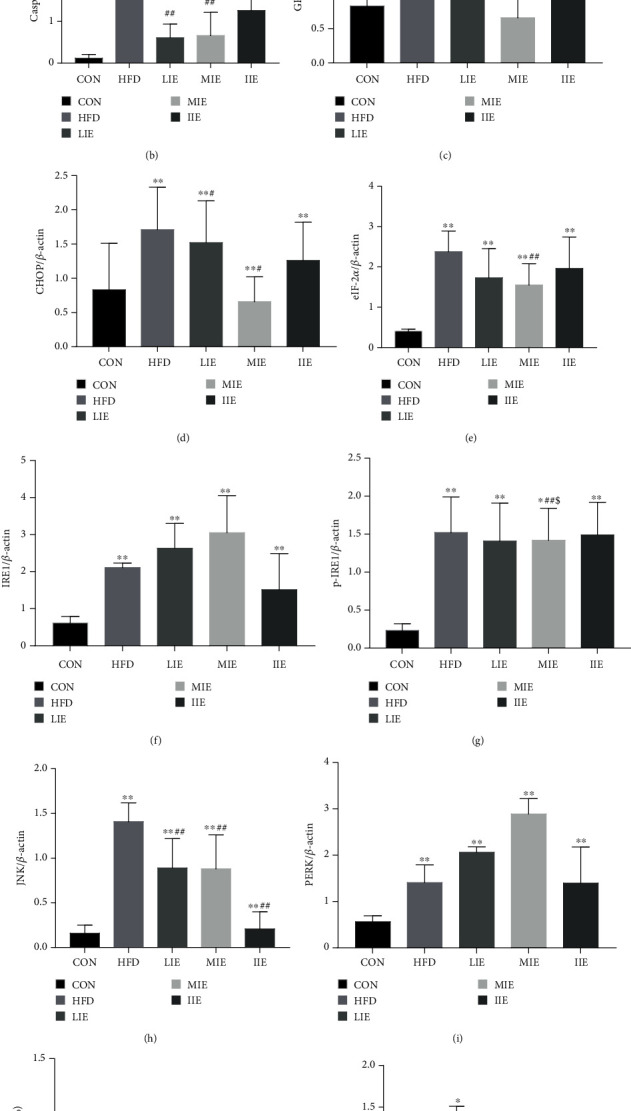
Western blot shows the proteins of ER stress. Representative Western blot analyses of ER stress protein (a) and the loading control (*β*-action), Caspase-3 (b), GRP78 (c), CHOP (d), eIF-2*α* (e), IRE1 (f), p-IRE1 (g), JNK (h), PERK (i), p-IRE1/IRE1 (j), and ATF4 (k). ^∗^*P* < 0.05 and ^∗∗^*P* < 0.01 vs. CON; ^#^*P* < 0.05 and ^##^*P* < 0.01 vs. HFD; ^&^*P* < 0.05 vs. IIE. All data are expressed as mean ± SD; 6–8 animals per group were used.

6 weeks of different exercise training intensities on the liver protein expression of GRP78, Caspase-3, PERK, eIF2*α*, CHOP, JNK, and the p-IRE1/IRE1ratio are shown in Figure 7. Western blot analysis indicated a significantly higher protein expression of CHOP, PERK, Caspase-3, eIF2*α*, JNK, and the p-IRE1/IRE1 ratio in the liver which were significantly increased by 12.4% (*P* < 0.01), 4.8% (*P* < 0.01), 10.3% (*P* < 0.01), 7.1% (*P* < 0.01), 11.2% (*P* < 0.01), and 1.8% (*P* < 0.05), respectively, in the HFD group after 6 weeks while GRP78 (*P* > 0.05) protein expression were not significantly identified in the HFD group compared with the CON group.

All exercise training intensity protocols did induce major effects on Caspase-3 (LIE vs. HFD, *P* < 0.05; MIE vs. HFD, *P* < 0.01; and IIE vs. HFD, *P* < 0.05) and CHOP (LIE vs. HFD, *P* < 0.05; MIE vs. HFD, *P* < 0.01; and IIE vs. HFD, *P* < 0.01), while eIF2*α* and the p-IRE1/IRE1ratio were downregulated only in the MIE group when compared with the HFD group (*P* < 0.01). In addition, PERK was significantly increased only in the IIE group in comparison with the HFD group (*P* < 0.01). Regarding GRP78, in all groups, no significant differences were found after the exercise training intervention. On the other hand, when we compared the levels of JNK between three exercise training protocols and HFD groups, we found no significant differences.

## 4. Discussion

NAFLD encompasses a spectrum ranging from simple steatosis to fatty liver with hepatocellular injury, nonalcoholic steatohepatitis, fibrosis, and cirrhosis [3]. The pathological mechanisms of NAFLD development are closely associated with primarily lipid metabolism disorders, low-grade inflammation, oxidative stress, and hepatocyte apoptosis [4, 5]. Exercise is a major component of treatment for NAFLD as recommended by the American Association for the Study of Liver Diseases [24], despite the fact that the optimal exercise intensity and mechanisms are undetermined [15, 16].

In this study, using a well-accepted HFD-induced NAFLD rat model [25], we found that rats fed with HFD for 16 weeks developed dyslipidemia, hyperglycemia, elevated expression of markers for the liver damage, and histology-based lipid metabolism disturbance. Because of failure of the hepatocyte to metabolize FFA excess, particularly long-chain saturated FFA, by converting them into TG, is associated with an increased risk for hepatocyte mitochondrial apoptosis pathway which mainly arises from FFA-induced oxidative stress of intracellular organelles, in particular the mitochondria and ER [8, 26]. For human research, Hashida et al. indicated a possible link between resistance exercise and lipid metabolism in the liver [27]. Keating et al. found no difference in efficacy of liver fat reduction by either aerobic exercise dose or intensity [12]. However, Abdelbasset et al. found that eight-week high-intensity interval aerobic exercise has a beneficial effect on IHTG, visceral lipids, and HRQoL in diabetic obese patients with NAFLD [28]. Indeed, accumulating evidence suggested that oxidative stress, lipid peroxidation, and oxidative damages to tissular and cellular molecules in humans play an essential role in the pathogenesis of NAFLD [29, 30]. In our study, we did not find any significant effect of HFD for 16 weeks on the CAT, GSH, and T-AOC activity being reduced; however, MDA concentration increased in the liver of the NAFLD rat model and SOD activity was significantly reduced, induced by 16 weeks of HFD.

Several researchers revealed that oxidative stress could induce ER stress and mitochondrial-mediated apoptosis. Indeed, upon ER stress, activated PERK leads to the phosphorylation of eIF2*α*, causing the global attenuation of translation and allows the preferential translation of UPR-dependent genes such as activation transcription factor 4 (ATF4) and proapoptotic effector CHOP (PERK/ATF4/CHOP pathway). As a common target of the other two canonical sensors of ER stress, activated IRE1 also promotes activation of apoptotic signaling kinase 1 which activates downstream stress kinase JNK that promotes apoptosis through inhibited and activated Bcl-2 and Bax [31], both of which are the two apoptosis-inducing substrates of p-JNK (IRE1/JNK pathway) [32]. In this study, we found that chronic high-fat diet may enhance the severity of NAFLD by enhancement of oxidative stress and activating ER stress (GRP78, PERK, eIF2*α*, CHOP, JNK, and the ratio of p-IRE1/IRE1) as well as the proapoptosis pathway (Bcl-2, which is an antiapoptotic protein, Bax, and Caspase-3) and apoptosis (TUNEL positive staining) in the liver. In agreement with that, Tanaka et al. reported that the IRE1/JNK pathway and PERK/ATF4/CHOP pathway were also upregulated simultaneously in association with apoptosis in livers of rats fed with 16 weeks of high-fat diet [21].

### 4.1. Effects of Exercise Training Intensity on Lipid Metabolism and Liver Injury

The present study did not find any improvements in HDL-c [33], but we did see improvements in TC, TG, LDL-c, and FFA concentrations after 6 weeks of all different exercise training intensities. In accordance with previously published data, which suggested that all exercise training intensities were effective at alleviating dyslipidemia as well as the hepatic damage.

Here, to study the effectiveness of exercise training intensity on the NAFLD outcomes, LIE, MIE, and IIE were introduced after the 16 weeks of high-fat diet. To date, several studies have shown improvement in lipid metabolism disorders following moderate-intensity exercise. There have been few studies to include three intensities of the exercise regimen for comparison which yielded different results. Consistent with our findings is that no sign was found different between the dose or intensity of the exercise regimen and reductions in FFA, TG, TC, LDL, ALT, and AST, which suggests that the sensitivity of TC responses to the exercise regimen seems to be higher than other serum NALFD markers. In addition, several recent studies have shown the impact of incremental-intensity endurance training compared to continuous moderate-intensity training on NAFLD outcomes [17, 34]. These results suggested that incremental-intensity exercise and moderate-intensity exercise were equally effective at lowering the serum FFA, TG, ALT, and AST of an HFD-induced NAFLD animal model. Moreover, a retrospective study also indicated that moderate- to vigorous-intensity physical activities yielded similar health benefits to those performing light-intensity physical activity and moderate to vigorous-intensity physical activity, in terms of the measured body adiposity and serum TG, ALT, and AST [35]. Winn et al. indicated that energy-matched high-intensity and moderate-intensity exercises are effective at decreasing intrahepatic lipid [36]. Collectively, we are the first to report that the LIE, MIE, and IIE were equally effective at alleviating dyslipidemia as well as the hepatic damage in HFD-induced NAFLD rat models; the results suggest that the therapeutic effect of exercise training against dyslipidemia and hepatic damage was in an intensity-independent manner.

### 4.2. Effects of Exercise Training Intensity on Oxidative Stress

Oxidative stress and those related to lipid peroxidation have been shown to impair nucleotide and protein synthesis in the liver, consequently resulting in apoptosis, inflammation, and fibrosis during the pathological condition of NAFLD development. Antioxidative stress effects induced by physical exercise may be important in terms of lifestyle management for NAFLD. Moreover, we report that to investigate the significance of 6 weeks, different exercise training intensities on oxidative stress in the livers of rats fed with 16 weeks if high-fat diet were done. The results of this study showed that only the MIE regimen effectively improves antioxidant capacity, as evidenced by the increased activities of T-AOC, SOD, and CAT, as well as altered levels of GSH; besides, MIE also induced a significant increase in SOD in comparison with the IIE and LIE groups, in which the results seem to suggest that the protective effect of exercise training against oxidative stress was in an intensity-dependent manner. Paradoxically, after 16 weeks of HFD regimen, the hepatic level reduction of MDA, which was a widely used oxidative stress biomarker, seems to be unrelated to exercise intensity. However, due to the lack of measurement of ROS and other related oxidative stress makers, the benefits of MIE in terms of antioxidative effects in an intensity-dependent manner could not be confirmed further in this study. Collectively, these data provide the first direct evidence of exercise intensity-dependent increased antioxidant levels in the livers of the NAFLD rat model. Concerning physical activity, the potent reduction in hepatic fat content and serum markers of oxidative stress levels in subjects who performed moderate to vigorous intensity may have contributed to improved NAFLD pathophysiology [35].

### 4.3. Effects of Exercise Training Intensity on Hepatocyte Apoptosis

The occurrence of hepatocyte apoptosis is mainly regulated by death receptor signal transduction pathways, mitochondrial signal pathways, and the ER pathway, which play a vital role in the progress of NAFLD to NASH, liver fibrosis, and even cirrhosis [37]. In the mitochondrial-driven apoptotic process, FFA excess and oxidative stress could increase Bax synthesis and move outside of the mitochondrial membrane, thereby, inducing the opening of the mitochondrial PT pore and the release of cytochrome C. On the other hand, Bcl-2 is mainly distributed in the outer mitochondrial membrane; its homodimer would suppress the opening of the PT pore, and the ratio of the Bcl-2/Bax protein complex is directly related to the occurrence of apoptosis. Therefore, exercise may also reduce apoptosis via reductions in the intrinsic pathway. The upregulation of the antioxidant defense system can also reduce oxidative stress-mediated apoptosis via the intrinsic pathway. Indeed, in agreement with that, Sinha et al. recently demonstrated that a high-fat diet upregulated proapoptotic and downregulated antiapoptotic enzymes, Bax and Bcl-2, respectively [38]. The present study showed that exercise training might reduce hepatocyte apoptosis in a rat model of NAFLD in an intensity-dependent manner. Our data showed that hepatocyte apoptosis was inhibited by MIE but not by LIE and IIE, which suggests that the distinct effect of three exercise regimens on hepatocyte apoptosis may be associated with its overall intensity. The previous study has been reported that both MIE (during 60 min/day at a starting speed of 15 m/min that was gradually increased until 25 m/min was reached) and voluntary physical activity decreased apoptotic (Bax content and Bax/Bcl-2 ratio) but only MIE was positively modulated. The present experiment showed that all the exercise intensity interventions significantly reduced the high-fat feeding upregulation of Bax and Caspase-3 and downregulation of Bcl-2 and the ratio of the Bcl-2/Bax in hepatocytes but the expression of the Bax protein significantly reduced in the LIE and MIE groups compared with the HFD group, which directly confirms intensity to be responsible for the observed protective phenotype. MIE was more effective at alleviating the reduction of the Bcl-2/Bax ratio than LIE and IIE.

### 4.4. Effects of Exercise Training Intensity on Endoplasmic Reticulum Stress

The exact mechanism by which exercise may reduce apoptosis in NAFLD remains to be elucidated. Taken together, our observation implies that high-fat diet leads to lipid metabolism disorders in NAFLD model rats, oxidative stress, the opening of apoptosis pathways (Bax, ATF4, and Caspase-3), leading to hepatocyte apoptosis, and further activation of endoplasmic reticulum stress proteins (GRP78, PERK, eIF2*α*, CHOP, JNK, and p-IRE1/IRE1). As Figure 5 shown that compared with the HFD group, the expression of Caspase-3 and JNK in all exercise groups significantly decreased, CHOP was decreased in the LIE and MIE groups, and the eIF2*α*, ATF4, IRE1, and the ratio of p-IRE1/IRE1 were only reduced in the MIE group. It is suggested that through the eIF2*α*/CHOP pathway, hepatocyte apoptosis was inhibited in the LIE and IIE groups. In contrast, MIE may inhibit IRE1/JNK1 and eIF2*α*/CHOP pathways, which control energy metabolism, inflammation, and lipid deposition of NAFLD model rats.

## 5. Conclusions

In summary, different-intensity exercise affected the reduction of dyslipidemia and liver injury in NAFLD rats but it was not related to the intensity of the exercise. Moderate-intensity exercise is more significant in improving the antioxidant ability and inhibiting hepatocyte apoptosis in NAFLD rats. The mechanism may be to reduce the expression of apoptosis-inducing Bax and ATF4 by increasing the Bcl-2 in liver tissue and mediate the IRE1/JNK and eIF2*α*/CHOP of the signal pathway of endoplasmic reticulum stress in hepatocyte.

## Data Availability

The data used to support the findings of this study are included within the article.

## Abbreviations

NAFLD: Nonalcoholic fatty liver disease
NASH: Nonalcoholic steatohepatitis
CON: Normal control group
HFD: High fat diet.
HFC: High-fat sedentary control
LIE: Low-intensity exercise
MIE: Moderate-intensity exercise
IIE: Incremental-intensity exercise
TG: Triglyceride
TC: Total cholesterol
HDL-c: HDL-cholesterol
LDL-c: LDL-cholesterol
FFA: Free fatty acid
SOD: Superoxide dismutase
MDA: Malondialdehyde
CAT: Catalase
GST: Glutathione
T–AOC: Total antioxidant capacity
CHOP: CCAAT enhancer-binding protein homologous protein
GRP78: Glucose-regulated protein 78
PERK: Protein kinase R-like ER kinase
eIF2*α*: Eukaryotic translation initiation factor 2*α*
IRE1: Inositol-requiring enzyme 1
p-IRE1: The phosphorylation of inositol-requiring enzyme 1, p-JNK, phosphor-c-Jun N-terminal kinases.
